# *Tgfβ2 *and *3 *are coexpressed with their extracellular regulator *Ltbp1* in the early limb bud and modulate mesodermal outgrowth and BMP signaling in chicken embryos

**DOI:** 10.1186/1471-213X-10-69

**Published:** 2010-06-21

**Authors:** Carlos I Lorda-Diez, Juan A Montero, Juan A Garcia-Porrero, Juan M Hurle

**Affiliations:** 1 Departamento de Anatomía y Biología Celular. Universidad de Cantabria/IFIMAV. Santander 39011. Spain

## Abstract

**Background:**

Transforming growth factor β proteins (Tgfβs) are secreted cytokines with well-defined functions in the differentiation of the musculoskeletal system of the developing limb. Here we have studied in chicken embryos, whether these cytokines are implicated in the development of the embryonic limb bud at stages preceding tissue differentiation.

**Results:**

Immunohistochemical detection of phosphorylated Smad2 and Smad3 indicates that signaling by this pathway is active in the undifferentiated mesoderm and AER. Gene expression analysis shows that transcripts of *tgfβ2 *and *tgfβ3 *but not *tgfβ1 *are abundant in the growing undifferentiated limb mesoderm. Transcripts of *tgfβ2 *are also found in the AER, which is the signaling center responsible for limb outgrowth. Furthermore, we show that Latent Tgfβ Binding protein 1 (LTBP1), which is a key extracellular modulator of Tgfβ ligand bioavailability, is coexpressed with *Tgfβs *in the early limb bud. Administration of exogenous Tgfβs to limb buds growing in explant cultures provides evidence of these cytokines playing a role in the regulation of mesodermal limb proliferation. In addition, analysis of gene regulation in these experiments revealed that Tgfβ signaling has no effect on the expression of master genes of musculoskeletal tissue differentiation but negatively regulates the expression of the BMP-antagonist Gremlin.

**Conclusion:**

We propose the occurrence of an interplay between Tgfβ and BMP signaling functionally associated with the regulation of early limb outgrowth by modulating limb mesenchymal cell proliferation.

## Background

Tgfβs constitute a subfamily formed in birds and mammals by 3 isoforms of secreted cytokines (Tgfβ1; Tgfβ2; Tgfβ3), which gives the name to the large Tgfβ superfamily made up of more than 30 structurally related proteins that comprises Activins, BMPs and GDFs. Tgfβs are multifunctional factors with important regulatory roles in adult and embryonic systems. During development Tgfβs are able to regulate almost all basic cellular processes including migration, proliferation, apoptosis and differentiation (reviewed by [1]). Their effects are mediated by binding to specific cell surface transmembrane receptors with serine/threonine kinase activity that trigger an intracellular cascade which regulates the expression of target genes and the biogenesis of specific microRNAs (reviewed by [2]). This basic signaling pathway is finely modulated by a large number of cofactors acting both at extracellular or intracellular levels which results in a variety of different responses depending on the lineage or the context of the target cells (reviewed by [3,4]). As a relevant example of this regulation, Tgfβs are secreted as latent precursor molecules covalently bound to latent Tgfβ-binding proteins (LTBP), which are components of the extracellular matrix. LTBPs act as a store for the cytokine but are also required for its activation [5].

Modulation of Tgfβs activity is also finely tuned at intracellular level. In the canonical signaling pathway, the activation of receptors results in phosporilation of Smad 2 and Smad 3 proteins which, upon binding with the adaptor Smad 4, translocate to the nucleus functioning as transcriptional regulators [3]. However, there are alternative and/or complementary intracellular pathways, like MAP kinases, activated in a cell-context dependent fashion. Furthermore, many intracellular regulators modify the pattern of gene activation/inhibition evoked by phosphorylated Smad proteins [6]. Hence, an appropriate functional characterization of these cytokines in the different systems will be improved by the identification of cofactors associated with the signaling pathway.

During limb development Tgfβs has been characterized as important regulators of the differentiation of the musculoskeletal system including cartilage, joint and tendon differentiation and morphogenesis [7-12] and myogenesis [13,14]. Possible roles in angiogenesis [15] and programmed cell death [16] have also been proposed. However, since the phenotype in mice with targeted alterations of this signaling pathway reflects large alterations of tissue differentiation and organogenesis of the musculoskeletal system [9-11], the possible implications of these cytokines during the initial stages of limb development have been largely neglected. Here we show that prior to the stages of tissue differentiation, *Tgfβ2 *and *Tgfβ3 *are coexpressed with *Ltbp1 *in the undifferentiated mesoderm and in the AER of the early limb bud. In addition, we provide evidence of a function of these factors in the regulation of mesodermal limb proliferation and in the modulation of the activity of *Gremlin*, a gene that is involved in the control of BMP signaling, a pathway of major importance in limb outgrowth and patterning.

## Results

### Phospho-Smad immunolabeling

**Figure 1:**
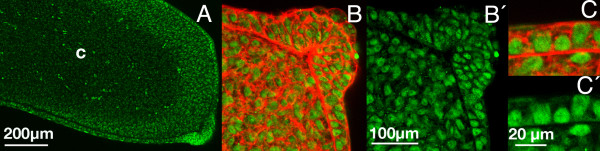
**pSmad 2 and 3 distribution during early limb development**. (**A) **Immunolabeling for p-Smad2 in limb buds at stage HH24 showing negative labeling of the central chondrogenic mesenchyme (c) in contrast with the subectodermal mesoderm and the intense positivity of the AER. (B) Detailed view of the distal tip of the limb bud at stage HH24 showing positive nuclear p-smad 3 immunolabeling (green) in the AER and distal mesenchyme contrasted with cytoplasmic phalloidin-TRITC labeling (red). B' shows only the green channel in B to see the positive nuclear labeling. (C) Detailed view of pSMAD3 nuclear labeling (green) counterstained with phalloidin (red). C' shows only green channel in C.

To explore the spatial distribution of Tgfβ-signaling, we examined the distribution of phospho-Smad 2 (p-Smad 2) and phospho-Smad 3 (p-Smad 3) by immunolabeling in embryonic limb tissue sections. As shown in Fig 1 nuclear labeling for both phospho-smads was intense in the AER and in the undifferentiated cells subjacent to the ectoderm and almost negative in the central chondrogenic region of the limb bud. Positive labeling of the undifferentiated mesoderm was highest in the distal mesoderm underlying the AER and more reduced at proximal levels of the bud, in the mesoderm located under the dorsal and ventral ectoderm. Importantly, the undifferentiated mesoderm underlying the AER, which is termed the "progress zone mesoderm" is responsible for outgrowth of limb along the proximo-distal axis.

### Expression of Tgfβs is coincident with regions of Smad 2, and Smad 3 activation

**Figure 2:**
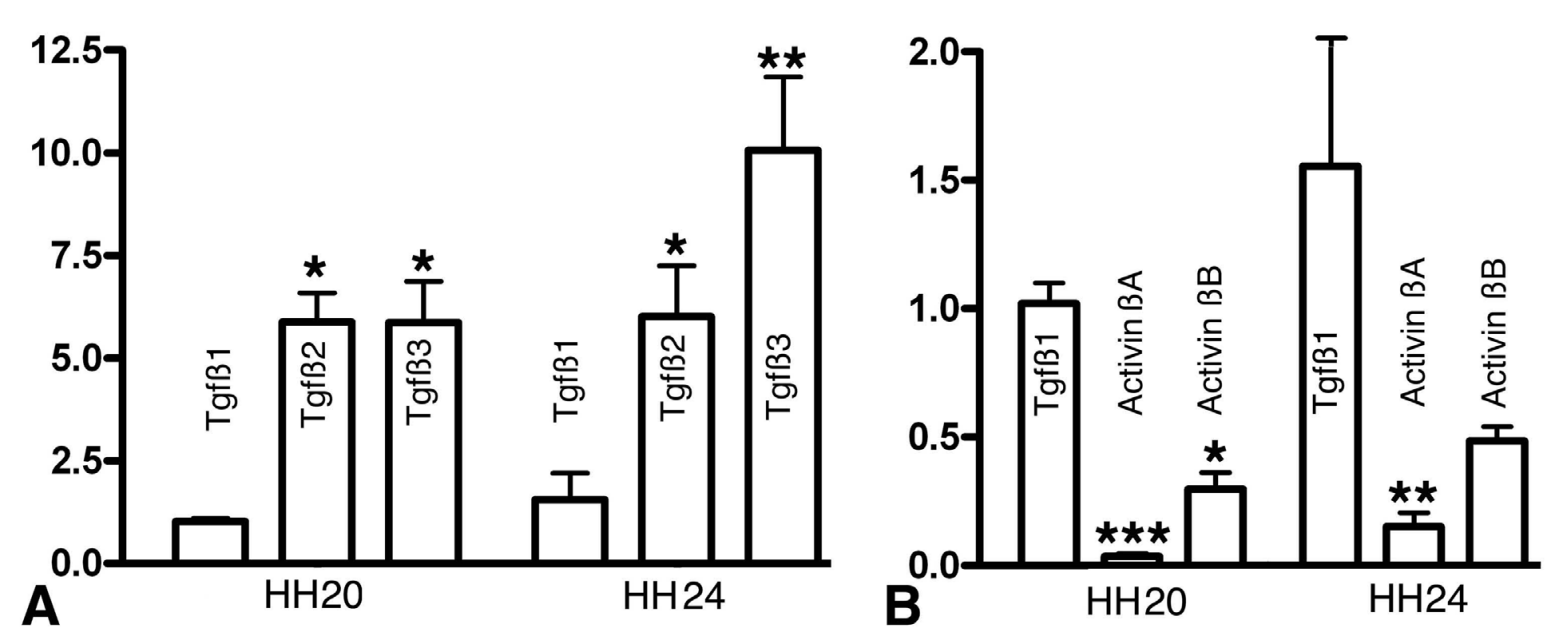
**Expression levels of different members of transforming growth factor β subfamily in the limb bud**. **(A-B) **Charts in A and B show the relative level of expression for the members of the Tgfβ subfamily (A) and for *Activin *subunits *βa *and *βb *(B). Note that *tgfβ1 *gene expression is the lowest of the three members of the family either at early limb bud stages (HH20; bars on the left in A) or at intermediate stages of limb development (HH24; bars on the right in A). (B) *Activin βa *and *Activin βb *displayed almost undetectable levels of expression by QPCR at the same stages of limb development. *Nodal *expression was totally absent and is not shown on the chart. All data was analyzed by real time QPCR and *tgfβ1 *expression at HH20 was used as calibrator. (*) p-value ≤ 0,05 or (**) p-value ≤ 0.01 or (***) p-value ≤ 0.001 using HH20 *tgfβ1 *expression level as calibrator.

Initial studies of Tgfβ gene expression by Q-PCR showed that Tgfβ2 and Tgfβ3 are the main components of this family expressed in the early developing limb and the number of transcripts increases as development proceeds (Figure 2A). In contrast Tgfβ1 (formerly Tgfβ4 in chicken) is expressed at very low levels. Expression levels of *Activins *and *Nodal *were also analyzed because these factors also activate Smad 2 and Smad 3 signaling. However, transcripts of *Activin beta *A and B subunits were almost undetectable (Figure 2B) and *Nodal *was absent (not shown). The pattern and intensity of gene expression of *Tgfβ *genes obtained by QPCR was similar in the early wing and leg buds (not shown), but domains of expression were better detected by in situ hybridization in the former.

**Figure 3:**
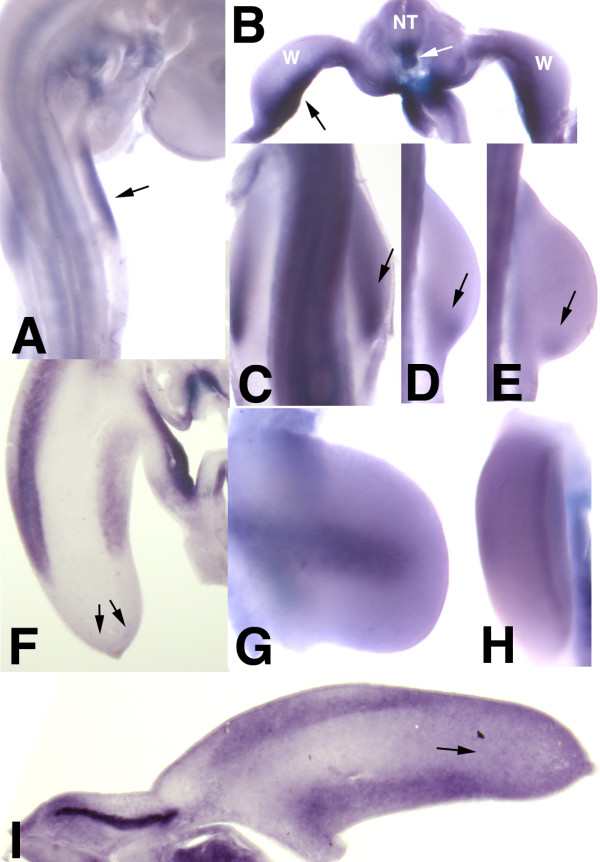
***Tgfβ *gene expression in the developing limb**. (A-H) Gene expression of *tgfβ2 *analyzed by in situ hybridization. (A) Whole mount in situ hybridization of an embryo at stage HH18 showing transcripts of *tgfβ2 *at the level of the limb field in the somatic mesoderm on the flank of the embryos (arrow). (B) Transverse vibratome section at the level of the wing bud (W) of an embryo at stage HH19 showing intense labeling in the somatopleura and associated limb mesenchyme (black arrow). Note also *tgfβ2 *transcripts in the floor plate of the neural tube (NT) and notochord (white arrow). (C-E) Detailed views of the wing bud at stages HH20 (C), HH21 (D), and HH22 (E) showing the expression domain of *tgfβ2 *in the mesenchyme of the posterior margin of the bud (arrows). (F-H) *Tgfβ2 *expression domains at stage HH24 are present in the dorsal and ventral mesoderm and in the AER. (F) Longitudinal vibratome section of the wing bud showing the intense accumulation of transcripts in the dorsal and ventral mesoderm associated with the premuscle masses, and a more reduced expression in the distal undiferentiated mesoderm (arrows). (G and H) are whole mount specimens illustrating the dorsal expression domain (G) and the AER domain (H). (I) Longitudinal section of the embryonic wing bud at stage HH24 showing the expression of *tgfβ3 *in the dorsal and ventral regions outlining the territory that is occupied by the premuscle masses. Note lower levels of expression in the undifferentiated distal limb mesoderm (arrow).

Transcripts of *Tgfβ2 *are first detected in the somatic mesoderm associated with the limb region at stage HH17-18 (Figure 3A). Next in development (stages HH19-22), transcripts are widespread through the limb mesoderm with domains of increased expression in the posterior and ventral regions of the bud (Figure 3B-E). By stages HH23 and HH24 expression is concentrated in the dorsal and ventral regions of the limb bud (Figure 3F-G). At these stages a mild but significant labeling is also observed in the AER (Figure 3H). From stage HH26 onwards labeling is associated with the developing digits and its expression pattern has been previously reported [12,17]. *Tgfβ3 *is expressed in the undifferentiated mesoderm of the early limb bud without specific domains until stage HH24, when transcripts accumulated in the dorsal and ventral mesoderm in the region occupied by the premuscular masses (Figure 3I).

Tgfβ1 expression by in situ hybridization is almost absent from the limb bud and lacks specific domains of expression.

### Expression and regulation of *LTBP *genes

**Figure 4:**
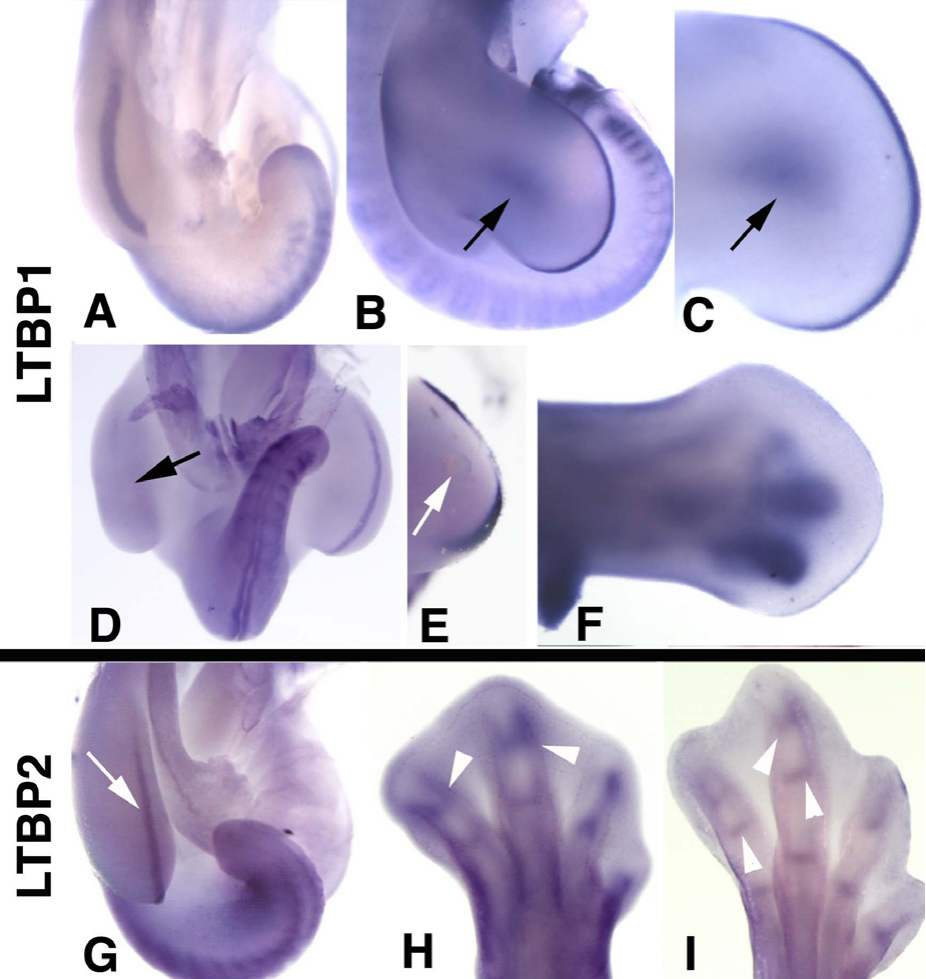
**Expression of *Ltbps *in the developing limb**. (A-F) Expression and regulation of *Ltbp-1*. (A) Whole mount in situ hybridization of *Ltbp-1 *gene showing intense labeling in the apical ectodermal ridge of the developing leg bud at stage HH20. (B-C) Whole-mount in situ hybridizations showing the expression of *Ltbp-1 *at stage HH24. Note that in addition to the AER domain, expression is also observed in the dorsal surface of the limb bud (arrows). (D) Image shows a dramatic down-regulation of *Ltbp1 *in the AER eight hours after the application of a BMP7- bead in the progress zone mesoderm (arrow). Compare the absence of labeling in the treated left limb with respect to the contralateral untreated control. (E) Whole mount in situ hybridization showing a more moderate downregulation of *Fgf8 *in the AER in a limb bud subjected to the same treatment to that shown in D (arrow shows the position of the BMP-bead). (F) Whole mount in situ hybridization showing the expression of *Ltbp1 *in the developing digits at stage HH26. (G-I) Expression of *Ltbp2 *in early (stage HH20; G) and late stages of limb development (stages HH30 and HH32; H and I). Note the expression domain in the AER of early limb buds (arrow in G) and the domains of the developing interphalangeal joints at the stages of digit morphogenesis (arrowheads in H and I).

To better characterize the signaling pathway activated by Tgfβs in the early limb bud we decided to study the expression of *LTBP*s as potential players of active Tgfβ delivery (Figure 4). In vertebrates there are 3 LTBP isoforms, namely LTBP-1, -3 and -4, which are able to bind and deliver Tgfβs [18] while the function of LTBP-2 remains uncertain [19]. We have studied by in situ hybridization the pattern of expression in the developing chicken limb of *Ltbp1*, *2 *and *3 *and all of them showed specific expression domains.

In the early limb bud, *Ltbp1 *is highly expressed in the AER (Figure 4A-B) and with much reduced intensity in the non-ridge ectoderm. By stage HH24 transcripts were also abundant in the dorsal mesoderm (Figure 4C). At later stages, expression of *Ltbp1 *was very intense in the condensing prechondrogenic aggregates of the developing digits (Figure 4F) where Tgfβs exert an important role in tissue differentiation [12]. Analysis of local gene regulation following local application of beads bearing BMP7, another well characterized regulator of limb development, showed an intense negative influence of BMPs on *Ltbp *gene expression in the AER (Figure 4D). This negative expression regulation was more intense than that induced in the markers of the AER, like *Fgf8 *(Figure 4E).

Prior to stage HH25, *Ltbp2 *was coexpressed with *Ltbp1 *in the AER (Figure 4G). At more advanced stages of development, *Ltbp2 *was expressed in the developing digits marking the zones of joint formation (Figure 4H-I).

*Ltbp3 *was not detected by in situ hybridization in the early limb bud. At more advanced stages *Ltbp3 *was expressed at very low levels in the differentiating phalangeal perichondrium and interphalangeal joints (not shown).

**Figure 5:**
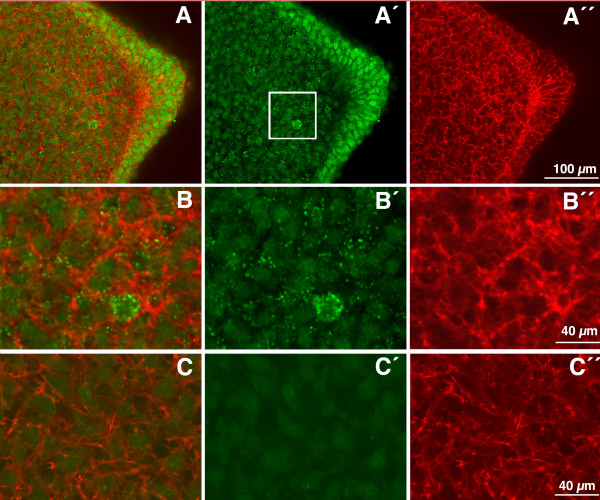
**LTBP1 immunolabeling in the early limb bud**. (A-B'') LTBP1 immunolabeling (green) counterstained with cytoplasmic phalloidin-TRITC labeling (red) of an early limb bud section (stage HH22) at the level of the AER. In all cases the merge images (A and B); the green channel for LTBP1 immunolabeling (A' and B'); and the red channel for actin labeling with phalloidin-TRITC (A'' and B'') are shown. (A-A") Note the strong labeling of the cells of the AER and the positive extracellular dotted labeling pattern in the underlying mesenchyme. (B-B'') detailed view of the region outlined by an square in A', showing the positive labeling of the matrix. Note the absence of overlapping between the cytoplasmic red labeling and the green spots indicative of its location in the pericellular space. (C-C'') Control section of a similar sample unexposed to the primary antibody for LTBP1.

In view of the spatial distribution of the *Ltbp1 *transcripts described above, we next analyzed by immunohistochemical approaches whether its protein distribution corresponded with regions of high Smad signaling. As shown in Figure 5 at initial stages of limb development, LTBP1 immunolabeling showed positive labeling in the ectodermal cells, with higher intensity in the AER (Figure 5A-A''). In addition a dotted labeling pattern was also appreciated in the mesodermal extracellular matrix (Figure 4B-B''), which correlated with the zones of intense p-Smad2 and 3 immunolabeling (see Figure 1). Neither the cellular nor the extracellular labeling was present in control sections unexposed to the primary antibody (Figure 5C-C'').

### Exogenous Tgfβs inhibit proliferation in limb bud explants

**Figure 6:**
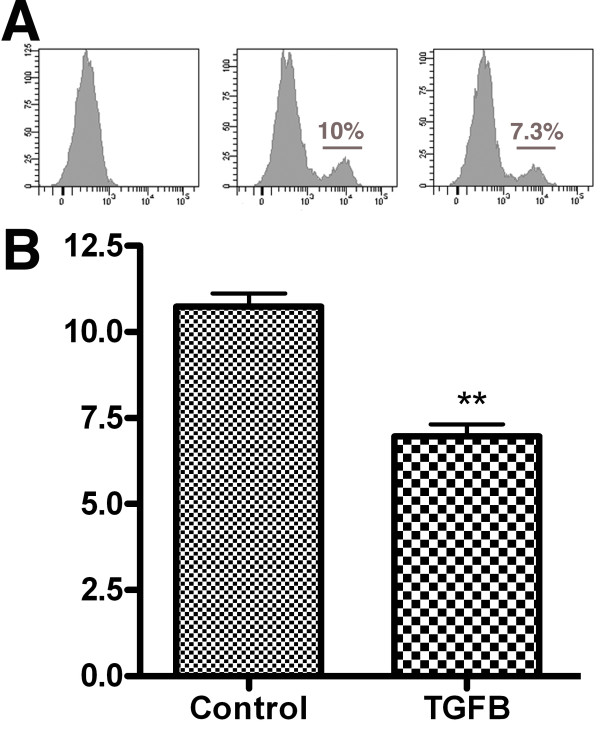
**Effect of Tgfβ treatments on mesodermal proliferation in early limb buds**. (A) Representative flow cytometry histograms to evaluate proliferation in limb bud explants of stage HH20/21 cultured for 18 hours in control media or in a medium supplemented with 0.033 μg/ml Tgfβ1 after labeling with Edu. (Left): control unlabeled sample; (middle): control sample untreated with Tgfβ; (right) Tgfβ treated sample. The percentage of labeled cells is indicated in the histogram. (B) Bars graph summarizing the rate of cell proliferation in control and treated limb explants.

Taking into account that Tgfβ signaling is active in the AER and progress zone mesenchyme prior to the onset of tissue differentiation we decided to explore the possible influence Tgfβ-signaling in the control of mesodermal cell proliferation. To check this potential function we set up cultures of the whole leg buds at stages HH20 or HH21 and mesodermal cell proliferation was evaluated by flow cytometry in untreated control explants and in explants cultured for 18 hr in presence of 0.033 μg/ml TGFβ1. As shown in Fig 6, cell proliferation in the treated explants was seen to undergo a 35% reduction in comparison with untreated cultures.

### Tgfβs regulate *Gremlin *gene expression in the early growing limb bud

**Figure 7:**
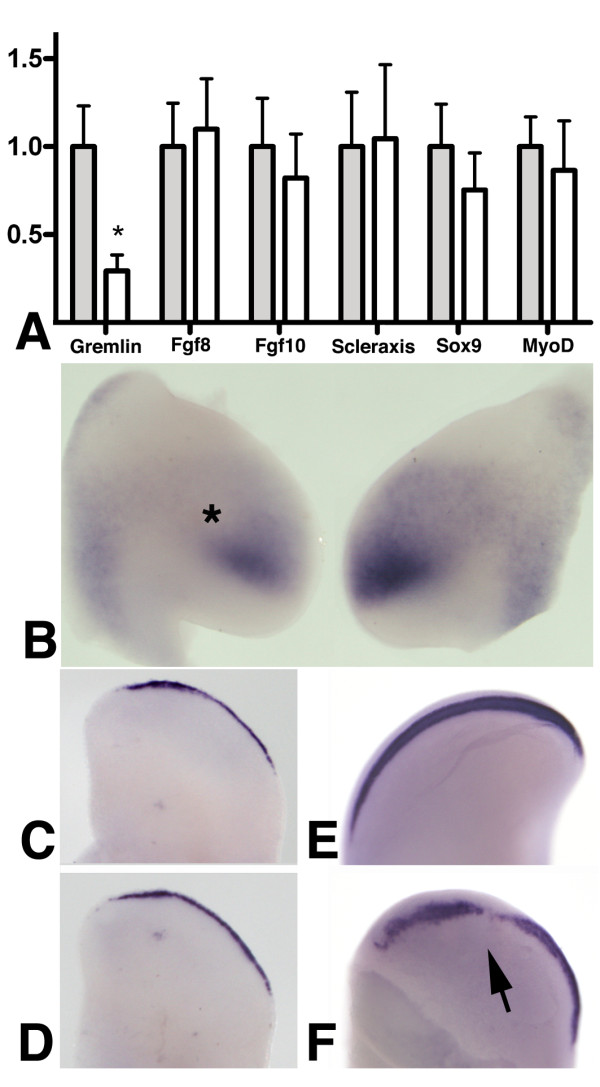
***Gremlin *expression is modulated by Tgfβ signaling in the early limb bud**. (A) Charts shows QPCR results for different genes from limb explants of stage HH20/21 cultured for 18 hours in control media (grey bars) or supplemented with 0.033 μg/ml Tgfβ1 (white bars). From left to right we show results for *gremlin, fgf8, fgf10, scleraxis, sox9 *and *myoD*. Each pair of bars shows control sample on the left and experimental situation on the right. The level of expression in controls was used as calibrator for each gene. Only *Gremlin *expression shows significantly lower levels of expression after Tgfβ treatments. (*) p-value ≤ 0,05 using control expression level as calibrator. (B) In situ hybridization showing down-regulation of *Gremlin *8 hr after the implantation of a Tgfβ1-loaded bead (*) in the right wing bud. (C-D) expression of Fgf8 in control (C) and Tgfβ treated limb explant (D) after 20 hr of culture. Note that expression is similar in control and experimental explants. (E-F) expression of Fgf8 in the AER of control (E) and experimental limb bud (F) 30 hr after the implantation of a Tgfβ-bead in the mesoderm subjacent to the AER (arrow). Note the irregularity of the expression domain in the experimental limb in comparison with the control untreated limb.

In view of the reduced proliferation observed in limb explants cultured in presence of Tgfβ we explored by QPCR changes in the expression of genes implicated in the control of limb outgrowth. In previous experiments we observed that neither *Scleraxis *nor *Sox 9 *or *MyoD *were regulated after 18 hr of culture in presence of TGFβs (Figure 7A), thus ruling out these cytokines having an effect inducing a precocious differentiation of the limb mesenchyme (see below). As markers for factors involved in the control of limb outgrowth we chose *Fgf8*, *Fgf10 *and the BMP antagonist *Gremlin*. Neither *Fgf8 *nor *Fgf10 *expression were significantly modified in the 18 hr time-period of culture chosen for these experiments, although the levels of expression of *Fgf10 *were always lower in the treated limbs (Figure 7A). In contrast, during the same time period *Gremlin *was intensely down-regulated (Figure 7A). This in vitro regulation of gene expression was also confirmed in vivo by in situ hybridization following the implantation of beads bearing TGFβ1 or TGFβ2 into the limb primordium (4 out of 5 experiments; Figure 7B).

Since Gremlin, by neutralizing BMPs, contributes towards maintaining the expression of *Fgf8 *in the AER [20], we next explored, by in situ hybridization, changes in the expression of *Fgf8 *in explants, or in the limb buds in vivo, exposed to TGFβ1 for longer time periods. Differences were not appreciated in explants during the first 24 hr of culture (Fig 7C-D) but a moderate downregulation of *Fgf8 *in the AER was appreciated in limbs 30 hr after the implantation of heparin beads bearing TGFβ1 (2 out of 5 experimental embryos; Figure 7E-F)

## Discussion

Our study provides evidence of the implication of Tgfβ signaling in early stages of limb development. The distribution of phosphorylated Smad 2 and 3 in the limb bud indicates that the AER, the distal undifferentiated mesoderm, and the dorsal and ventral mesoderm are zones of remarkable signaling activity, while the differentiating central core mesoderm at these stages is almost negative. According to our expression analysis *Tgfβ2* and *Tgfβ3* are the two members of this family accounting at these stages for active signaling in the developing limb, while *Tgfβ1 *and other members of the Tgfβ superfamily signaling through Smad 2/3 (Activins and Nodal) are almost absent. We have also observed that the zones of active signaling correlated closely with the domains of expression of *Ltbp1*, which is an important extracellular regulator of the signaling pathway. In physiological conditions, Tgfβs are secreted as inactive complexes consisting of Tgfβ cytokine, a N-terminal latency associated peptide (LAP) and Latent Tgfβ binding protein (LTBP; [5]). Studies in a variety of systems have shown that LTBP regulates the bioavailability of Tgfβs facilitating its secretion, storing the inactive Tgfβ within the extracellular matrix, and, regulating its activation in the pericellular space (see [21,22]). Accordingly, Tgfβ activity appears decreased in mice harboring mutations of *Ltbp *genes (see [23]). Consistent with these functions we have observed that domains of expression of *Ltbp1 *correlate closely with the zones of high p-Smad 2 and 3 immunolabeling.

A considerable number of studies have previously addressed the role of Tgfβs in limb development. From these studies Tgfβ signaling is currently associated with regulation of chondrogenesis, tenogenesis, myogenesis and joint formation [9-14]. Here we observed that mesodermal proliferation is significantly reduced in limb bud explants cultured in presence of Tgfβs. These findings are consistent with the recognized antiproliferative effects of Tgfβ in other systems [24], but the precise mechanism responsible for such reduced mesodermal cell proliferation following treatments with Tgfβs remains elusive. The absence of up-regulation in the expression of *Scleraxis*, *Sox 9 *and *MyoD*, which are major regulators of tendon, cartilage and muscle differentiation respectively, contrast with the intense regulation of these factors observed at more advanced stages of limb development [11,12,25]. This finding rules out the posibility that inhibition of proliferation was secondary to the initiation of mesodermal cell differentiation and reveals a different stage-dependent response of the limb mesoderm to this signaling pathway. We have also observed that expression of *Fgf8 *and *Fgf10*, which are key factors in the control of limb outgrowth [26,27], is not modified in short term treatments with Tgfβs. Consistent with our findings, up-regulation of Tgfβ signaling has been previously proposed as being responsible for reduced limb outgrowth in different experimental approaches [28,29], however, mutants with defective Tgfβ signaling tend to exhibit limbs of reduced rather than elongated size [10]. In the case of Tgfβ2-deficient mice, which is the predominant member of the family expressed at early stages of limb development, the limbs appear grossly deformed and abnormally rotated accompanied by a reduced size of the zeugopodial skeleton of the forelimb [30]. Hence, the effect on cell proliferation detected in our study does not appear to be reflected in the phenotype of mice with defective Tgfβ-signaling. It is likely that this influence in proliferation might be obscured in the course of development as Tgfβs at more advanced stages of development are abundantly expressed in the growth plate of long bones and participate in the control of chondrocyte differentiation, proliferation, matrix synthesis, and mineralization [31].

Studies on a variety of developing systems have provided evidence of a functional interplay between Tgfβ- and BMP-signaling. A good example of these interactions is observed in the growth plate of postnatal mice. In this system chondrocyte maturation involves a coordination between negative and positive effects of Tgfβs and BMPs respectively [31]. In the developing limb the formation of the cartilaginous primordia of the digits appears to also be regulated by the local interplay between both signaling pathways [32]. Here we have shown that Tgfβ-signaling has a negative influence in the expression of *Gremlin*, a secreted BMP antagonist that has a key function in the maintenance of limb outgrowth [20,33,34]. The physiological function of Gremlin is to counteract the negative effects of BMPs on the AER [35,36] and its downregulation at the end of limb morphogenesis causes the termination of limb outgrowth [37]. Altogether these findings suggest the occurrence of a negative interactive loop between Tgfβs and BMPs implicated in the regulation of limb outgrowth. However, additional and/or alternative growth regulatory mechanisms cannot be ruled out, as the decreased mesodermal limb proliferation caused by Tgfβ treatments was detected prior to the down-regulation of *Fgf8 *in the AER. Another possibility, which cannot be ruled out, is that the interaction between Tgfβs and BMPs was also associated with the recent demonstrated function of Tgfβs modulating the response of early limb mesodermal cells to BMP-signaling (see [38]).

## Conclusions

The possible role of Tgfβs in early limb bud development has been poorly studied to date. Here we have characterized the expression of the different *Tgfβs *and the Tgfβ extracellular regulators *Ltbps *in this model. The expression in the Apical Ectodermal ridge and undifferentiated mesoderm, together with the pattern of activation of the intracellular Tgfβ canonical pathway mediated by Smads transcription factors, indicate a possible role of these cytokines modulating limb bud outgrowth. Indeed we further show that Tgfβs reduce cell proliferation in the undifferentiated limb mesenchyme. Concomitant with this role we find that BMP and TGFβ signaling pathways establish a cross-regulation of their modulators Ltbp1 and Gremlin respectively during early limb bud development.

## Methods

In this work, we employed Rhode Island chicken embryos ranging from 2,5 to 5 days of incubation corresponding to stages 18 to 32 of Hamburger and Hamilton (HH).

### In vivo experimental manipulation of the limb

Eggs were windowed at the desired stages and experimental manipulations of the limb were performed in the right leg bud using forceps to handle the embryo and membranes. Local treatments were performed by application at the desired regions of heparin (Sigma) or Affi-Gel blue (BioRad) beads incubated for 1 hour in 5 μg/ml Tgfβ1, 5 μg/ml Tgfβ2, (R&D Systems) or 0,5 mg/ml BMP7 (a gift of Creative Biomolecules, Hopkinton, MA). After manipulation, the eggs were sealed and changes in gene expression were analyzed by in situ hybridization

### Explant cultures

Limbs of stages HH20 or HH21 embryos were sectioned using iridotome and placed on 0.4 μm Culture Plate Insert Millicell (Millipore) for further culture in DMEM (100 units/ml penicillin and 100 μg/ml streptomycin). In experimental explants 0.033 μg/ml Tgfβ1 were added to the medium. After 18-20 hr of culture samples were processed for mRNA extraction and QPCR analysis.

### Flow cytometry

Limb explants of stage HH20/21 cultured for 18 hours in control media or in a medium supplemented with 0.033 μg/ml Tgfβ1 were dissociated to single-cell level in order to perform flow cytometry analysis based on direct DNA labeling of proliferating mesodermal cells using 5-ethynyl-2'-deoxyuridine (EdU) chemistry [39]. The EdU labeling was performed using the Click-iT EdU Alexa Fluor 488 Flow Cytometry Assay Kit (Invitrogen) according to the manufacturer's instructions. Each sample consisted of 8 limb buds.

### Antibodies, immunolabeling and Confocal Microscopy

The following primary polyclonal rabbit antibodies were used: phospho-Smad2 (Ser465/467; Cell Signaling); phospho-Smad3 (Cell Signaling); and LTBP1 (Santa Cruz signaling). Actin staining using 1% or Phalloidin-TRITC (Sigma) was performed to mark cytoplasm and enhance nuclear or extracellular labeling. For immunolabeling samples were fixed in 4% paraformaldehyde and sectioned 100 μm thick in a vibratome. Samples were examined with a laser confocal microscope (LEICA LSM 510) by using a Plan-Neofluar 10×, 20× or Plan-Apochromat 63× objectives, and an argon ion laser (488 nm) to excite FITC fluorescence and a HeNe laser (543 nm) to excite TRITC.

### Probes and in situ hybridization

In this study we used probes for *tgfβ2 *[8], *tgfβ3 *[8], *gremlin *[40], *fgf8 and fgf10*, (kindly provided by Cliff Tabin). In addition, fragments of chicken *tgfβ1*, *ltbp1*, *ltbp2 *and *ltbp3 *genes were obtained by RT-PCR. First-strand cDNA was synthesized with random hexamers and M-MulV reverse transcriptase (Fermentas) and 1 mg of total RNA from day 7 autopods. The following primers were used for subsequent PCR amplification: for ***tgfβ1 ***5'-tcttcgtgttcaacgtgtcc-3' and 5'-cgcagcagttcttctcatcc-3'; for ***ltbp-1 ***5'-tgcatcaaacctaactgtgca-3' and 5'-tcggaagttagtggctgtca-3'; for ***ltbp-2 ***5'-agatccacctggatgtctgc-3' and 5'-ctcacagccattgagaatgc-3' and for ***ltbp-3 ***5'-attcggaggagcagagc and 5'-tggcagtggcagttgtagg -3'.

The PCR conditions were 94°C, 4 min and then 35 cycles of 94°C, 20 s; 60°C, 30 s; 72°C, 60 s; and final extension at 72°C, 10 min. PCR products were subcloned into pGEM T-easy (Promega). Sequencing of the probes was performed to verify specificity. Digoxygenin-labeled sense and antisense RNA probes were generated for in situ hybridization analysis.

In situ hybridization of control and treated limbs was performed in 100 μm vibratome sectioned specimens. Samples were treated with 10 μg/ml of proteinase K for 20-30 minutes at 20°C. Hybridization with fluorescein or digoxigenin labeled antisense RNA probes was performed at 68°C. Alkaline phosphatase-conjugated anti-digoxigenin antibody (dilution 1:2000) was used (Roche). Reactions were developed with BCIP/NBT substrate as the chromogene (Roche).

### Real time quantitative PCR (Q-PCR) for gene expression analysis

In each experiment total RNA was extracted and cleaned from specimens using the RNeasy Mini Kit (Qiagen). RNA samples were quantified using a spectrophotometer (Nanodrop Technologies ND-1000). First-strand cDNA was synthesized by RT-PCR using random hexamers, the M-MulV reverse transcriptase (Fermentas). The cDNA concentration was measured in a spectrophotometer (Nanodrop Technologies ND-1000) and adjusted to 0.5 μg/μl. Q-PCR was performed using the Mx3000P system (Stratagene) with automation attachment. In this work, we have used SYBRGreen based QPCR. *Gapdh *had no significant variation in expression across the sample set and therefore was chosen as the normalizer in our experiments. Mean values for fold changes were calculated for each gene. Expression level was calculated relative to a calibrator according to the 2^-(ΔΔCt) ^equation [41]. Each value in this work represents the mean values and SEM of at least three independent samples obtained under the same conditions. Each sample consisted of 4 limb buds. Data were analyzed using one-way ANOVA followed by Bonferroni tests for post-hoc comparisons between expression levels of Tgfβs, and Student-t-test for gene expression levels in the treated developing limbs. Statistical significance was set at p < 0.05. All the analyses were done using SPSS for Windows version 15.0. Primers for QPCR were: for ***sox9 ***5'-gaggaagtcggtgaagaacg -3' and 5'-gatgctggaggatgactgc -3'; for ***scleraxis ***5'-caccaacagcgtcaacacc -3' and 5'-cgtctcgatcttggacagc -3'; for ***fgf8 ***5'-cgtgttcatgcacttgttcg -3' and 5'-gatctgtcaccaggctctgc -3'; for ***fgf10 ***5'-atcgagaagaacggcaagg -3' 5'-ggacttaactgccacaactcc -3'; for ***tgfβ1 ***5'-acctcgacaccgactactgc -3'and 5'-cttccactgcagatccttgc -3'; for ***tgfβ2 ***5'-tgcactgctatctcctgagc -3' and 5'-gcatgaactgatccatgtcg -3'; for ***tgfβ3 ***5'-ctcagtggcaggaatgtgc -3'and 5'-cgaggttggactctctgtgc-3'; and for ***gremlin ***5'-agtcgcaccattatcaacagg -3'and 5'-ttgcagaaggaacaagactgg -3'; for ***nodal ***5'-ccaagaagtacaacgcctacc -3'and 5'-gcatgtacgcgtgattgc -3'; for ***activin βA ***5'-gctgactgtccatcatgtgc -3'and 5'-actgcttccaccatctcagg -3'; for ***activin βB ***5'-tggatcatagcaccatcagg -3'and 5'-gcattcggtacttgattcacg -3'; ***myoD ***5'-acggcatgatggagtacagc -3'and 5'-tccgtgtagtagctgctgtcg -3'.

## Authors' contributions

CILD and JAM performed the largest part of the experiments and participated in the design of the study, documentation, evaluation and interpretation of data and in the writing of the manuscript. JAGP collaborated in some experiments and in the design of the study. JMH participated in the design of the study, evaluation of results and interpretation of data, and wrote the manuscript. All authors read and approved the final manuscript.
